# Subtype frequency, demographic features, treatment and outcome of Juvenile Arthritis in one Centre in Abu Dhabi in the United Arab Emirates

**DOI:** 10.1186/s12969-023-00796-w

**Published:** 2023-02-07

**Authors:** K. Khawaja, R. Kalas, N. Almasri

**Affiliations:** 1 Paediatric Rheumatology, Pediatrics, Sheikh Shakhbout Medical City, Abu Dhabi, United Arab Emirates; 2 Division of Internal Medicine, Sheikh Shakhbout Medical City, Abu Dhabi, United Arab Emirates; 3 Division of Pediatrics, Sheikh Shakhbout Medical City, Abu Dhabi, United Arab Emirates

**Keywords:** Children, Juvenile idiopathic Arthritis (JIA), Outcome, Abu Dhabi

## Abstract

**Background:**

Juvenile Idiopathic Arthritis is a chronic inflammatory disease that affects 1 in 1000 children worldwide. Our population in the United Arab Emirates is diverse.

The objective of this study is to describe the subtype frequency, demographic features and treatments received and outcome of our patients.

**Methods:**

Patients with the diagnosis of Juvenile Arthritis identified through the hospital electronic medical records system (EMR), which was implemented for all medical documentation in January 2011. All patients included are patients who presented to our center for treatment and follow up from 2011 to end of 2021. Retrospective case notes review of patients electronic medical records with the diagnosis of JIA was performed.

**Results:**

One hundred thirty-eight patients in total. Oligoarticular subtype was the most represented with 75 patients (55%) followed by Rheumatoid factor negative polyarticular JIA with 32 patients (23%) then Enthesitis related arthritis (ERA) with 10 patients (7%) then psoriatic (6%) then systemic JIA (5%). Undifferentiated subtype of 2%.

The most diagnostic delay is in enthesitis related arthritis subtype with a mean of 11.4 months (6–25) followed by undifferentiated JIA with a mean of 7.5 months (4–8.5).

131 (96%) out of 138 received steroid treatment. Only 6 patients did not receive steroids.

Out of 138 patients, 101 (73%) were on synthetic disease modifying medication methotrexate. Sixty-eight patients out of the total 138 required biologic treatment (49%).

In total 93 patients achieved clinical remission (67%). In remission on treatment 78 patients which is (56%) of the total number of patients with follow up ranging from 1 to 5 years and 84% of patients in remission. In remission off treatment 15 patients (11% of all patients and 16% of patients in remission).

**Conclusion:**

The most common subtype in our cohort of patients is oligoarticular JIA. Longest delay is for ERA subtype.

All our patients with oligoarticular JIA received Intra articular steroid injection as first line treatment. 49% of our patients received biologic treatment similar to rate in Northern Europe. Our remission rate is 67% with 11% of patients are in remission off treatment.

Access to care remains a priority to treat patients effectively.

## Introduction

Juvenile Idiopathic Arthritis (JIA) is a chronic inflammatory disease that affects 1 in 1000 children worldwide [1]. It is the most common cause of chronic arthritis in children. It is a heterogeneous group of autoimmune conditions classified into seven different subtypes, based on the clinical features in the first 6 months of presentation as per the International League of Associations for Rheumatology (ILAR) classification system [2–4]. There is limited access to pediatric rheumatology worldwide. Our population in the United Arab Emirates (UAE) is diverse. The total population is 9,680,000. 42% of the population is Emiratis and other Arabs. 50% is South Asian. 8% is Western and other [5, 6]. The number of children under 14 years of age is 1.49 million (boys 0.76 million and girls 0.73 million) [7]. The population of Abu Dhabi is 2.3 million. In this paper we are aiming to focus on the subtype frequencies, demographic data, treatment and remission rate in our cohort of patients with JIA. Our center in Sheikh Shakhbout Medical City is in the capital city of Abu-Dhabi. Referrals to the center are received through the public and private sector. All residents of Abu Dhabi hold health care insurance. Not all residents outside Abu Dhabi hold health care insurance coverage, but the majority do have insurance cover.

The primary objective of this study is to describe the subtype frequency and demographic features of our patients and the secondary objective is to look at the treatments received and the outcomes of our patients.

## Methods

Patients with the diagnosis of JIA have been identified through the hospital electronic medical records system (EMR), which was implemented for all medical documentation in January 2011. Using the International Statistical Classification of Diseases and Related Health Problems (usually referred to by the shorter name “International Classification of Diseases (ICD)), version 9 (ICD 9) (ICD 9 code 714, 714.30, 714.89, 696) and ICD 10 (M08.0), we sought out all patients under 16 years of age with the diagnosis of JIA classification as per International league of Arthritis and Rheumatism diagnostic criteria and classification (ILAR) [2]. All patients included are patients who presented to our center for treatment and follow up. A retrospective case notes review of patients electronic medical records with the diagnosis of JIA was performed. Patients older than 16 years of age at diagnosis were excluded. Patients not diagnosed with JIA were also excluded.

We looked at the demographic data of all patients included in the study. Age at onset, age at diagnosis and duration from onset to diagnosis, ethnic back ground, sub type of arthritis as per ILAR classification, ANA, RF and anti CCP result, HLA B27 typing, treatment received and outcome.

The outcomes have been assessed through clinically inactive disease as per the wallace criteria which includes; no joint with active arthritis, no fever or rash or serositis or splenomegaly or lymphadenopathy attributed to arthritis, no active uveitis, ESR or CRP or both if both done within the normal range, the physician's global assessment of disease activity as lowest possible. Clinical remission with medication was defined as inactive disease for 6 consecutive months while on medication, and clinical remission without medication was defined as inactive disease for 12 consecutive months or more while the patient is off all medications [8–10].

We also used the clinical Juvenile Arthritis Disease Activity Score (cJADAS 10) for disease remission of score equal or less than 1 and Juvenile Arthritis Damage Index (JADI) articular and JADAI extra articular > 0 for long term outcome [11–14].

The study was approved by the hospital ethics committee.

Simple descriptive statistics were used to describe the results. Statistical analysis using the SPSS software package for Windows version 25.

## Results

We carried a retrospective review from 2011 to end of 2021. The total number of patients included in the study was 138 new and follow up patients. The prevalence of JIA for our cohort of patient was 32 /100,000. With regards to subtype distribution; 66 patients had oligoarticular JIA (48%). Nine had extended oligoarticular JIA (7%). Thirty-five patients had polyarticular (25%), 7 patients had systemic JIA (5%). 10 patients had enthesitis related arthritis (7%) 8 patients had psoriatic arthritis (6%) and 3 patients had undifferentiated arthritis (2%).

There were 85 Emirati patients in total and 28 patients from other other Arab nationalities with total of 113 patients which is 82% of the total cohort of patients. Eleven children were of Asian origin (8%) and 9 children were of Western origin (6.5%) and 5 children were of African origin (3.5%) (Table 1).

**Table 1:** Patient ethnicity

Ethnicity	Number of patients	%
Emirati	85	62
Middle East	28	20
Asian	11	8
Western European/American	9	6.5
African	5	3.5

The median age at disease onset was 4 years (IQR 2 to 8 years). Median disease duration is 6 years (IQR 3–7 years).

Ninety-eight patients are female (71%) and 40 patients are male (29%). Female patients represent 85% of persistent oligoarticular, 89% in extended oligoarticular, 67% in RF positive and 62% in RF negative JIA. 57% of ERA and systemic JIA and 75% of psoriatic JIA patients.

The most diagnostic delay is in enthesitis related arthritis subtype with a mean of 11.4 months (6–25) followed by undifferentiated JIA with a mean of 7.5 months (4–8.5). Oligoarticular JIA with delay of 4.2 months (3–5.6). For polyarticular JIA mean diagnostic delay of 4 months (3–5) for RF negative and 5 months (4.2–6) for RF positive JIA. Systemic JIA was diagnosed in 2 months in all patients (Table 2).

**Table 2:** Subtype frequencies, gender, age and Diagnostic delay

JIA subtype	Number of patients (%)	Gender(M/F) N/N (%/%)	Age at Dx Years	Diagnosis Delay (months ±SD)	Entry cJADAS 10 (range)
Persistent Oligo	66 (48)	10/56 (15/85)	6 (1–14)	5.4 (3–5.6)	11 (9–15)
Extended Oligo	9 (7)	1/8 (11/89)	4 (2–13)	3.2 (3–5.6)	
Poly RF + ve	3 (2)	1/2(33/67)	15 (13–16)	5 (4.2–6)	19 (17–22)
Poly RF-ve	32 (23)	12/20 (38/62)	7.5(3–13)	4 (3–5)	19 (17–22)
ERA	10 (7)	3/4(43/57)	9(8–16)	11.4 (6–25)	14 (13–16)
Psoriatic	8 (6)	2/6(25/75)	6 (5–15)	1 (1–5)	13 (12–15)
Systemic	7 (5)	3/4(43/57)	2 (1–6)	1.2 (1–2)	21 (19–24)
Undifferentiated	3 (2)	1/2	8 (6–10)	7.5 (4–9)	12 (11–13)
Total	138 (100)	40/98 (71/29)			

*JIA* Juvenile Idiopathic Arthritis, *ERA* Enthesitis Related Arthritis. CJADAS 10

ANA was positive in 10 patients (7%), RF was positive in 3 patients (2%), HLA-B27 was positive in 5 patients (3.6%), Anti CCP was positive in 4 patients (3%), ESR was in normal range 1–20 mm/hr. in 15 patients and was raised in 123 patients (89%), mildly raised 20–40 mm/hr. in seventy patients who were all oligoarticual. ESR was moderately raised 40–60 mm/hr. mainly in polyarticular, psoriatic, oligoarticular and ERA. Significantly raised > 60 mm/hr. in ten patients (all systemic JIA and 3 of the polyarticular subgroup). CRP was raised in 24 patients (17%). Ninety out of the 138 patients had x-ray and USS looking for erosions with ten having evidence of erosions on x-ray or USS. All allocated on their hands.

131 (96%) out of 138 patients received steroid treatment. All Oligoarticular JIA patients received joint injections with triamcinolone hexa acetonide (90%) or triamcinolone acetonide (10%). In total the patients who received joint injections were 82 patients (59%) of the total patients. Remission was achieved in 58% of the persistent oligoarticular JIA. Twenty-eight patients were lost to follow up, out of these 25 patients were followed up for one year and remained in remission during this time. Eighty-six children required oral steroids (62%). Twenty children required intravenous systemic steroids (15%). All systemic JIA patients (7) received Intra venous Methyl prednisolone 30 mg/kg maximum 1 g followed by oral prednisolone 1 mg to 1.5 mg daily, maximum of 20 mg tapered over two to four weeks. Biologic treatment was introduced as needed. Thirteen poly articular JIA patients with a significant delay in presentation received IV methylprednisolone 10-15 mg/kg 3 doses over three days followed by oral prednisolone 1 mg/kg for average of 3 weeks (range of 1 to 4 weeks). An earlier start of biologics was achieved for all subtypes from 2019.

Out of 138 patients, 101 (73%) were on synthetic disease modifying medication methotrexate. Seventy patients (69% of the total patients on methotrexate) received sub cut methotrexate and 31 (31%) received oral methotrexate. Sixty-eight patients out of the total 138 required biologic treatment (49%). Of these 45 patients are Emirati and 23 are from other ethnic backgrounds. 41 (33%) patients received etanercept 43 patients recieved adalimumab (35% of total patients on biologics). 24 patients received tocilizumab (21 intravenous and 3 patients on sub cut) 18% of total patients on biologics, 5 patients received abatacept (4% of total patients on biologics), 5 patients received anakinra (4% of total patients on biologics),  4 patients received canakinumab (3% of total patients on biologics), 2 patients received rituximab (1.6% of total patients on biologics) and another 2 patients received Infliximab (Table 3).

**Table 3:** Number of patients on different types of biologic agents

Biologic	Number of Patients	%
Etanercept	41	33%
Adalimumab	43	35%
Tocilizumab	24	20%
Abatacept	5	4%
Canakinumab	4	3%
Anakinra	5	4%
Rituximab	2	1.60%
Infliximab	2	1.6%
Total	126	

In total 93 patients achieved clinical remission (67%). In remission on treatment 78 patients which is (56%) of the total number of patients with follow up ranging from 1 to 5 years and 84% of patients in remission. In remission off treatment 15 patients (11% of all patients and 16% of patients in remission). Active disease in 10 patients (7.2% of all patients) and lost to follow up 35 patients (25%), out of these 28 patients (80%) have persistent oligoarticular JIA subtype who completed their treatment and did not come back for follow up after 1 year (Table 4).

**Table 4:** Outcome of patients with JIA subtypes

JIA subtype	Active	In remission	Remission on treatment	Remission without treatment	Lost to FU/Discharged	Total number of patients
Persistent Oligo	0	38	24	14	28	66
Extended Oligo	0	8	7	1	1	9
Poly RF + ve	1	2	2	0	0	3
Poly RF-ve	2	25	25	0	5	32
ERA	2	8	8	0	0	10
Psoriatic	2	5	5	0	1	8
Systemic	2	5	5	0	0	7
Undifferentiated	1	2	2	0	0	3
Total	10	93	78	15	35	138

*JIA* Juvenile Idiopathic Arthritis, *ERA* Enthesitis Related Arthritis

In regards to subtypes and remission rate; persistent oligoarticular JIA in remission 58% of total persistent oligoarticular patients. Twenty-eight patients are lost to follow up. We looked back and 25 out of the 28 followed for 1 year following treatment then discharged. For extended 89% in remission. For RF negative polyarticular JIA; 25 out of 32 are in remission which is 78%. All on treatment, 5 lost to follow up and 2 still have active disease. For enthesitis related arthritis 8 (80%) out of 10 patients in remission on treatment and the rest still have disease activity. For psoriatic 5 (63%) are in remission; of these 4 in remission on treatment and one patient lost to follow up. For systemic 5 (72%) out of 7 patients in remission on treatment and 2 (29%) still have disease activity on treatment.

### Composite disease activity score cJADAS10 of 2.0 (IQR 0–20)

JADI articular > 0 in 4 patients (2.9%) and JADAI extra articular > 0 in 3 patients (2.1%) due to Uveitis and cataract. JADAI total > 0 in 7 patients 5%.

17 patients had Uveitis (12.3%). Out of these 12 had oligo articular JIA and 5 poly articular JIA. 2 had cataract. 2 patients have evidence of joint erosions.

A total of 23 patients transitioned to adult care; out of these 8 patients transitioned to our adult rheumatologists and 15 transitioned to other adult rheumatologists either in the private or outside our city.

## Discussion

In our study although the numbers are small, the prevalence for our cohort is slightly higher to our neighbouring country Oman which was reported in 2015 to be 20/100,000 [15]. We need a country wide study to better understand the epidemiology of JIA in our population. In regards to subtype distribution, the most frequent subtype is oligoarticular JIA of 48%. This supports the findings in the International experience in the EPOCA study by Consolaro et al. [16]. But the representation of the Middle Eastern and African subcontinent was limited in this study. Another study that was done in Oman in 2015, the commonest subtype was RF-ve polyarticular JIA followed by oligoarticular JIA. Our finding of oligoarticular subtype being the most common also resembles the findings from English and Canadian cohorts [17, 18] (Table 5). The latest review of epidemiological literature of JIA in the Middle Eastern region by Al-Mayouf et al. highlights that there is not enough literature describing JIA in this region. Only 8 journal publications were identified concerning epidemiology and 42 articles describing JIA subtypes from Africa and Middle East [19, 20]. The literature from Asia, especially India and Taiwan; the commonest subtype is enthesitis related arthritis. There is also studies from Turkey describing ERA as the commonest subtype [17, 18, 21–24]. Our population is diverse with Asian ethnic back ground representing 50% of the overall population. Although the majority of these are young migrant workers, some are with families and children. There could be under referral of children with possible ERA that would explain the predominance of oligoarticular subtype rather than ERA which is what is expected for our population from the available literature worldwide. There is possibly a number of patients with juvenile arthritis being cared for by doctors in the private sector including pediatricians, adult rheumatologists and possibly orthopaedic surgeons especially of early ERA presenting with back pain. Also sometimes the difficulties of accessing specialised care might there could also be lack of awareness amongst professionals of juvenile arthritis generally but more so of ERA subtype and children with back pain which would explain the under referral of patients. Juvenile arthritis is an exclusion diagnosis representing a phenotypically heterogeneous group of conditions of unknown aetiology. Genetic basis is not fully understood and can be of more significance in geographical areas were consanguinity is common. Further genetic studies are needed especially from our area to better understand the genetics which would influence the classification criteria [25, 26].

**Table 5:** Frequency of JIA subtypes by Geographical Area. (%)

JIA subtype	Our Study	Oman^a^	Turkey^b^	India^c^	Taiwan^d^	England^e^	Canada^f^	Africa/ Middle East^g^
Oligoarticular	54.3	31.8	32.9	20.8	23.1	45.9	40.7	37.8
Poly RF + ve	2	7.5	3.8	11.9	4.6	2.3	3.7	5
Poly RF-ve	23	39.2	13.5	17.4	11.8	13.4	20.2	22.4
ERA	7	3	32.9	35.7	37.4	6.3	10.1	9.2
Psoriatic	6	0.9	1.9	1.2	1.5	6.9	6.5	3.1
Systemic	5	17.8	13.2	8.0	19	5.3	7.3	16.9
Undifferentiated	2		1.9	4.6	2.6	3.9	11.5	5.6

^a^Abdwani R. Pediatric Rheumatology. 2015;13:33

^b^Çakan M. J Pediatr. 2017;59 (5):548–554

^c^Kunjir V. J Rheumatol. 2010 Aug 1;37 (8):1756–62

^d^Shen CC. J Microbiol Immunol Infect. 2013 Aug;46 (4):288–94

^e^Adib N. Rheumatology (Oxford). 2008 Jul;47 (7):991–510

^f^Oen K. Arthritis Rheum. 2009 Aug;15;61 (8):1077–86

^g^Al-Mayouf SM. Pediatr Rheumatol Online J. 2021 Dec 2;19 (1):166

In regards to diagnostic delay; the most delay is in enthesitis related arthritis subtype with a mean of 11.4 months followed by undifferentiated subtype with a mean of 7.5 months. This supports the argument in subtype distribution of ERA being under represented and the most delay in diagnosis. We believe there is significant diagnostic delay in ERA and patients could be presenting later in to our adult rheumatology colleagues as adults with advanced ankylosing spondylitis or enthesitis related arthritis [8].

Triamcinolone hexa acetonide is available in the UAE. It is the steroid used for most of our patients needing joint injections as it is the preferred steroid with better outcome for children [27]. Eighty-two patients received joint injections which is all the Oligoarticular subtype as initial treatment or as treatment of disease flare. Also a small percentage of polyarticular patients who needed joint injections to joints that did not have full response to systemic treatment. There was more use of systemic steroids for patients presenting before 2019. Since then there has been easier access to biologics with the publication of the new ACR guidelines [28, 29].

Sixty-eight patients (49%) received biologic treatment. Out of these 55 patients of Emirati ethnic back ground and 23 of other ethnic back ground. 64.7% out of the total Emirati patients required biologic treatment and 43.4% of Non-Emirati patients required biologic treatment. There is more Emirati patients with polyarticular JIA and patients from same extended families with more significant disease. Non Emirati patients had more oligoarticular JIA. This highlights that access to care remains the more important factor. Access to biologic treatment was appropriate once the child is referred and diagnosed.

Our percentage of patients receiving biologics is higher than what is published recently in the literature for this region in the world experience paper by Consolaro in 2019 [16]. they reported 24.4% of the patients receiving biologics from Africa and Middle East. Also higher than what is reported for Northern America and Eastern and Southern Europe in the same paper but similar to Northern Europe. This could be explained by the fact that our patients present later with more significant disease requiring more aggressive treatment. Also the limited access to care with milder cases possibly getting managed by adult rheumatologists and pediatricians. We tend to get patients with more aggressive disease. Our remission rate of patients off medications is 11%. This could also reflect that our patients have more significant disease with 56% of our patients are in remission but continue to be on medication. 33% of our patients still have active disease compared to 46% of the Netherlands cohort with active disease (mild end) [30, 31]. This difference could be explained by the higher percentage of our patients receiving biologics. Also our under reported number of patients with ERA which tend to have more active disease needing continuing treatment. Our higher percentage of patients in remission on medication reflects the number of patients on biologics.

We are aware of the limitations of our paper. This is a retrospective review so there will be degree of bias. Our lost to follow up is high with 25% of the total patients but the majority (28 out of 35 which is 80%) is oligoarticular JIA patients who had been well for 6 months. This could be patients in remission not requiring further medical care or non-Emirati patients returning to their home country of origin.

The delay in referral of patients has remained an important factor for our cohort as patients receive the required treatment once they can access care. Access to biologic treatment can go through some delay when there is inadequate health insurance cover but we have managed to identify funding for all patients in need of treatment. There is still need to increasing awareness of juvenile arthritis presentations especially potential ERA subtype in a child.

## Conclusion

The most common subtype of the current JIA classification in our cohort of patients is oligoarticular JIA. There is still limited access to care and need to increase awareness and referrals especially for ERA subtype which might have been underrepresented.

All our patients with oligoarticular JIA received Intra articular steroid injection as first line treatment. 49% of our patients received biologic treatment similar to rate in Northern Europe. Our remission rate is 67% with 11% of patients are in remission off treatment. There is low rate of damage with JADI > 0 in 7 patients (5%). A priority for us remains to be access to care in order to treat our patients effectively. 

## Data Availability

The datasets from this study available on request from corresponding author.

## Abbreviations

JIA: Juvenile idiopathic Arthritis
ERA: Enthesitis Related Arthritis
ILAR: International league of Arthritis and Rheumatism
UAE: United Arab Emirates
JADAS: Juvenile Arthritis Disease Activity Score
JADI: Juvenile Arthritis Damage Index
ANA: Anti-Nuclear Antibody
RF: Rheumatoid factor
CCP: Cyclic citrullinated peptide
HLA: Human Leukocyte Antigen
